# Five-Year Retrospective Analysis of Eurosilicone’s Silicone Gel–Filled Breast Implants

**DOI:** 10.1093/asjof/ojz018

**Published:** 2019-06-20

**Authors:** Maurizio Saturno, Sharon Stewart, Erin Bell, Emanuela Esposito

**Affiliations:** 1 Plastic Surgery Unit, St Carlo Hospital, Potenza, Italy; 2 GC Aesthetics, Glasgow, UK; 3 Breast Unit, Department of Clinical Medicine and Surgery, University of Naples Federico II, Naples, Italy and the Department of Breast Cancer, Istituto Nazionale Tumori Napoli – IRCCS – Fondazione G. Pascale, Naples, Italy

## Abstract

**Background:**

Silicone breast implants have been widely used for breast augmentation and reconstruction. During this time, silicone breast implants have undergone several modifications to improve their safety, quality, and clinical performance. Complications such as reoperation, capsular contracture, and rupture are risks often associated with breast implants.

**Objectives:**

The authors conducted a retrospective study to analyze and report complication rates associated with Eurosilicone’s (Eurosilicone S.A.S, Apt, Cedex, France) silicone gel–filled breast implants over a period of 5 years.

**Methods:**

In this retrospective clinical study, 2151 women who underwent either breast augmentation or breast reconstruction with Eurosilicone breast implants were diagnosed. Data on early and late complications including implant removal (explantation/exchange), capsular contracture, and rupture were collected using questionnaires, completed by 39 surgeons across Italy.

**Results:**

Of the 2151, only 60 patients (2.78%) required implant removal. Twenty-five patients experienced capsular contracture (Baker Grade III/IV), giving an actual rate of 1.2%. The actual rate of implant rupture confirmed by breast magnetic resonance images was 0.18% (4 implants). Six patients (0.27%) were diagnosed with breast cancer following breast augmentation, and local complications including hematoma (1 patient) and seroma (2 patients) were experienced.

**Conclusions:**

This retrospective clinical study involving Eurosilicone’s round and anatomical textured silicone gel–filled mammary implants demonstrates an excellent safety profile through 5 years.

**Level of Evidence: 2** 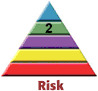

Silicone gel–filled breast implants have been commercially available for over 50 years.^1^ During this time, breast implants have undergone a number of modifications to improve their safety, quality, and clinical performance.^2^ Despite the introduction of newer techniques such as lipomodeling,^3^ breast implants continue to be the standard for breast augmentation^4^ as breast implant surgery is the most popular plastic surgery procedure performed.^4,5^

In 2010, a breast implant manufacturer known as PIP (Poly Implant Prosthèse, La Seyne-sur-Mer, France) was banned from manufacturing and selling breast implants after it was discovered that PIP implants contained an industrial-grade silicone gel. This gel is known to contain higher levels of contaminants than medical-grade gel and have a higher than normal incidence of rupture. Following this, surgeons throughout Italy refused to use silicone breast implants manufactured by French companies for a period of time. Thus, the safety and effectiveness of Eurosilicone’s silicone gel–filled breast implants are now being evaluated. Herein we present the results of a retrospective 5-year clinical study on the complication rates associated with Eurosilicone’s silicone gel–filled breast implants on 2151 patients who underwent primary breast augmentation, revision breast augmentation, and breast reconstruction.

## METHODS

**Figure 1.**
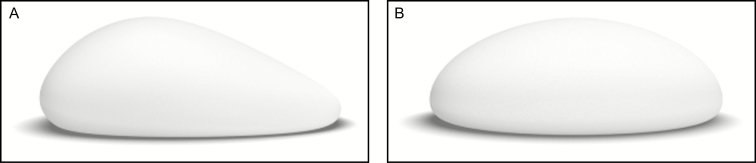
Photographs of Eurosilicone’s breast implants. (A) Anatomical textured mammary implant. (B) Round textured mammary implant. Permission was obtained from Eurosilicone S.A.S. to publish these images.

Eurosilicone’s mammary implants are round and anatomical, textured silicone gel–filled implants which received their CE mark in 1997. These implants are manufactured using medical-grade silicone from NuSil Technologies, an ISO 9001–certified supplier and incorporate a 360° Paragel barrier layer. Eurosilicone’s breast implants comply with the European Medical Device Directive 93/42/EEC as amended 2007 and this study complies with the Declaration of Helsinki. Figure 1 provides an illustration of the implants under examination.

This retrospective analysis examined the safety of Eurosilicone’s silicone gel–filled breast implants for primary augmentation, revision augmentation, and reconstruction.

The study was conducted over a 5-year period from 2008 to 2013, collecting data from 39 surgeons across Italy.

All patients who underwent breast surgery with a Eurosilicone breast implant were included (2151 patients). Written informed consent was obtained from all patients. Data collection began at the first consultation, including patient’s history and demographics. Following surgery, patients returned for physical examinations conducted by their surgeon twice in the first year and annually thereafter to 5 years.

Eurosilicone initiated a scheme known as the Ultimate Patient Satisfaction Program (UPSP), whereby in the event of a complication such as air bubbles in the implant, implant rupture (confirmed by breast imaging), capsular contracture Baker Grade III/IV, seroma, and cosmetic dissatisfaction, Eurosilicone would provide replacement implants free of charge. To receive replacement implants, the surgeon had to complete a complaint form shown in Supplementary Appendix A. This provides Eurosilicone with customer information, details of the device including serial number and lot number, operative details, and reason for the complaint. In case of implant rupture, surgeons were asked to return the damaged implant and breast magnetic resonance images to Eurosilicone for analysis. Once the presence of a complication was assessed, Eurosilicone provided the surgeon with replacement breast implants. Eurosilicone collected and provided data in compliance with Legislative Decree 196/03. All complications experienced were documented on the complaint form and sent to Eurosilicone to review. These complications will be reported throughout.

The goal of this retrospective study was to determine the rate of postoperative complications through 5 years. The primary complications measured were reoperation, followed by capsular contracture and implant rupture. Local complications including hematoma, seroma, infection, autoimmune disease, and breast cancer were also documented. The Kaplan-Meier method was utilized to determine the cumulative risk of reoperations and other complications. MINITAB 17 software was used for statistical analysis.

Before enrolment into this study, patients were screened against inclusion and exclusion criteria. All patients who underwent breast surgery with a Eurosilicone implant were screened. The inclusion criteria outlined that all subjects must be genetically female; must have undergone one of the following procedures: primary breast augmentation, revision breast augmentation or breast reconstruction; must be willing to attend all required follow-up visits; and provide informed consent and willing to undergo magnetic resonance imaging (MRI) evaluation. The exclusion are patients who had declared pregnancy or nursed a child within 6 months; had a history of abscesses or infections; were incapable of providing informed consent owing to mental disorder or had any condition that impedes the use of MRI should be excluded from the study. All patients were implanted in accordance with the manufacturer instructions for use.

## RESULTS

### Surgical Characteristics

Of the 2151 patients, 1986 (92.3%) underwent primary augmentation, 86 (4.0%) underwent revision augmentation, and 79 (3.7%) patients had breast reconstruction (Table 1). All breast reconstruction patients underwent tissue expansion followed by permanent breast implant insertion at least 6 months post-mastectomy. The median age was 37 years (age range, 18–65 years). All patients received round or anatomical silicone gel–filled mammary implants manufactured by Eurosilicone. Round implants were used in more than 95% of patients. The median implant volume was 310 cm^3^ (range, 100–500 cm^3^); details are provided in Table 2. The most common incision site utilized for breast augmentation was peri-areolar, followed by the inframammary fold incision. Submuscular implant placement was mainly performed during primary breast augmentation.

**Table 1.** Initial Indication for Surgery

Primary augmentation	Secondary augmentation	Primary reconstruction
1986 (92.3%)	86 (4.0%)	79 (3.7%)

**Table 2.** Volume Distribution of Eurosilicone’s Mammary Implants

Implant volume range (cc)	No. of implants	Percentage of implants	Cumulative percentage of implant volumes
100-150	100	2.3	2.3
200-240	300	7.0	9.3
260-280	1176	27.3	36.6
300-325	1339	31.1	67.8
350-375	1075	25.0	92.7
400-450	232	5.4	98.1
500-550	80	1.9	100.0

### Reoperation

Over a period of 5 years, 60 out of 2151 patients required reoperations, which resulted in an explantation or exchange of implants across all cohorts. The actual rate for surgical revision was 2.78%. The reasons for reoperation included unsatisfied cosmetic result (20 patients), capsular contracture (25 patients), implant rupture (4 patients), bubbles (2 patients), seroma (2 patients), hematoma (1 patient), and breast cancer (6 patients). Further details can be found in Table 3.

**Table 3.** Reasons for Reintervention Throughout 5 Years

Adverse event	1st year	2nd year	3rd year	4th year	5th year	Total
Unsatisfied cosmetic outcome	6	5	5	3	1	20
Capsular contracture	–	8	7	6	4	25
Implant rupture	4	–	–	–	–	4
Bubbles	–	2	–	–	–	2
Seroma	2	–	–	–	–	2
Haematoma	–	–	1	–	–	1
Cancer	1	1	2	1	1	6

### Implant Rupture

When a suspected rupture was identified, the patient would undergo MRI to confirm the diagnosis. Across 5 years, a total of 4 patients experienced implant rupture which required surgical reintervention, giving an actual rupture rate of 0.18%. All ruptures were spontaneous.

### Capsular Contracture

Capsular contracture Baker Grade III/IV was reported to have occurred 25 times throughout this 5-year clinical study. There were no reports within the first year; however, this jumped to 8 by year 2, a further 7 patients experienced this complication in year 3, 6 in year 4, and a final 4 in year 5. The actual rate of capsular contracture at 5 years was 1.16%. The Kaplan-Meier estimated survival rates are provided in Table 4. This analysis revealed that there was no risk of developing capsular contracture Baker Grade III/IV within the first year post-implantation. This percentage increased steadily over the next 4 years; 0.37% for the second year, 0.70% for the third year, 0.99% for the fourth year, and 1.18% after 5 years.

**Table 4.** Kaplan-Meier Risk of Developing Capsular Contracture Across All Cohorts

Time interval	Cumulative probability of failure	Standard error		95% confidence interval	
		Lower (%)	Upper (%)	Lower	Upper
0	1	0.00	0.00	0.00	0.00
1	2	0.37	0.13	0.12	0.63
2	3	0.70	0.18	0.35	1.06
3	4	0.99	0.22	0.57	1.41
4	5	1.18	0.24	0.72	1.64

### Local Complications

Many local complications may occur as a result of the surgical procedure itself and not due to the presence of the implants. Throughout this 5-year retrospective study, the rate of local complications was particularly low.

Infection is a complication associated with any surgical procedure and not just breast surgery. Therefore, it is interesting to highlight that, of the 2151 patients, no one experienced an infection which required surgical intervention. This could be due to advanced surgical technique. In addition, there were no patients who developed an autoimmune disease across the 5-year period.

Another complication often associated with surgery and is a common complication following breast implantation is a hematoma. In this series of patients, just 1 patient reported this complication, giving an overall actual rate of 0.05%.

Two women experienced early seroma within 5 years post-implantation. The actual rate of developing seroma across all cohorts was 0.1%.

Within 5 years, breast cancer was newly diagnosed in 6 women. All cases were reported in patients within the primary or revision augmentation cohorts. The actual rate for developing breast cancer at 5 years was 0.27%. Neither new primary breast cancer nor local recurrent breast cancer occurred among patients in the breast reconstruction cohort.

Furthermore, no cases of anaplastic large cell lymphoma were reported. However, this is expected due to the time to diagnosis for this complication being 9 to 10 years and this study having a follow-up period of just 5 years.

**Figure 2.**
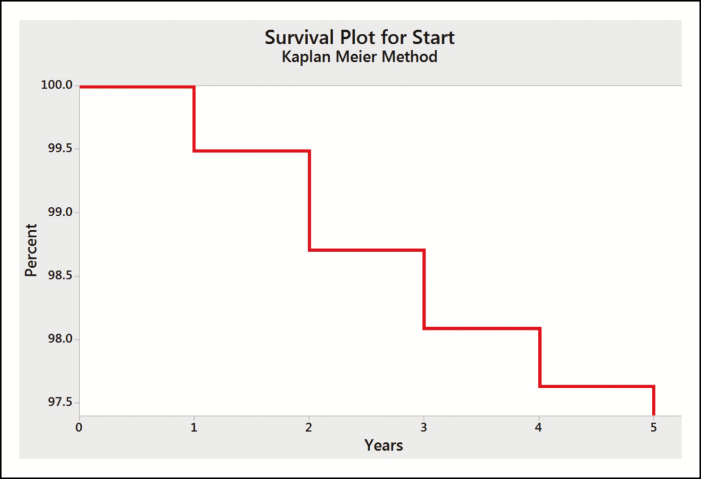
Five-year Kaplan-Meier curve illustrating the survival plot for breast implants.

All complications excluding capsular contracture were grouped in a statistical analysis cumulative flair. Table 5 provides the results of this analysis with the cumulative average of an adverse event occurring at each year of this study (0.51%, 1.30%, 1.91%, 2.37%, and 2.60%, respectively). Figure 2 shows breast implant life expectancy across the 5-year study using the Kaplan-Meier curve. The Kaplan-Meier curve confirms the increased risk of an adverse event occurring after year 2, although the risk remains particularly low. From the third year onwards, the risk of occurring a complication decreased progressively.

**Table 5.** Odds Ratio for Surgery Failure Through Five Years

Time interval	Cumulative probability of failure	Standard error		95% confidence interval	
		Lower (%)	Upper (%)	Lower	Upper
0	1	0.51	0.15	0.21	0.81
1	2	1.30	0.24	0.82	1.78
2	3	1.91	0.29	1.33	2.48
3	4	2.37	0.33	1.73	3.01
4	5	2.60	0.34	1.93	3.28

## DISCUSSION

Breast augmentation and breast reconstruction using silicone implants are the most common procedures performed by plastic surgeons.^6^ This 5-year retrospective clinical study presented results which demonstrate the low complication rates associated with Eurosilicone’s silicone gel–filled mammary implants, which received their CE mark in 1997. There are many complications associated with surgery in general which include but are not limited to infection, hematoma, seroma, and pain. Conversely, complications observed specifically in breast surgery include implant rupture, reoperation, and capsular contracture. This study retrospectively examined 2151 patients who received round or anatomical textured Eurosilicone breast implants and the results successfully highlight the low complication rates at 5 years post-implantation.

The objective of this study was to demonstrate the safety of Eurosilicone’s gel-filled mammary implants through the reoperation, capsular contracture, and rupture rates in both augmentation and reconstruction patients. Survival probabilities over a 5-year period were calculated using the Kaplan-Meier estimator and from the Kaplan-Meier survival curves the likelihood of any adverse event to occur after the second year post-surgery was observed.

It should be highlighted that there are differences in the study design and patient population making it difficult to make direct comparisons. The total reoperation rate was 2.78% through 5 years, which is in line with other manufacturer’s results at 5 years.^7,8^ The reoperation rate for this study is significantly lower than Natrelle’s (Allergan, Inc., Irvine, CA) 6-year core study on their Style 410 implants.^8^ They reported an actual reoperation rate of 34.6%. Furthermore, the capsular contracture rate within this study was reported to be 1.16%, which is lower than competitor’s results. Sientra (Sientra, Inc., Santa Barbara, CA) reported a capsular contracture rate across all cohorts of 7.0% at 5 years post-implantation.^9^ In this retrospective study, the actual rate of capsular contracture at 5 years was extremely low, and even lower than the Kaplan-Meier estimates.

Finally, the rupture rate within this study is particularly low at 5 years post implants with an actual rate of 0.2% for all cohorts. These data on a large patient population are in line with the available literature and demonstrate an excellent safety profile of silicone gel–filled breast implants manufactured by Eurosilicone.

Our confidence in Eurosilicone’s breast implants is further supported by Duteille et al, who published 8-year results on the safety and performance of Eurosilicone’s textured, Cristalline Paragel mammary implants.^10^ Duteille et al prospectively collected clinical data on 526 women at 17 centers across France. The results at 8 years yielded low complication rates. The Kaplan-Meier risk of implant rupture across all cohorts was 1.4% per patient and 0.9% per implant. The risk of capsular contracture (Baker Grade III/IV) per implant was 8.4% in the primary augmentation cohort and 18.0% in the primary reconstruction cohort. The actual implant removal rate was 6.0% and 13.8% for breast augmentation and breast reconstruction, respectively. The incidence of local complications including infection and seroma was 0.6% and 0.2% by subject, respectively. Results from this multicenter study demonstrate the safety and effectiveness of Eurosilicone breast implants through 8 years.

It is important to highlight the main limitation of this study is its retrospective design. It would typically be recommended for a long-term prospective clinical study to be conducted to confirm the results of this study. However, Duteille et al conducted a long-term study on Eurosilicone’s implants and the results of this study are comparable with the results in this retrospective analysis.

## CONCLUSION

The results presented in this 5-year retrospective clinical study demonstrate the high safety profile associated with Eurosilicone’s round and anatomical, textured mammary implants for breast augmentation and reconstruction. The data provide surgeons with additional information on the medium-term complication rates associated with these implants and show the results are in line with competitor brands. Additional long-term clinical studies are required and the introduction of worldwide breast implant registries is warranted to compare results consistently.

## Disclosures

Dr. Saturno has joined the UPSP but has no financial interest with Eurosilicone S.A.S. Dr. Stewart and Ms. Bell are employees of GC Aesthetics, the parent company of Eurosilicone. Dr. Esposito declared no potential conflicts of interest with respect to the research, authorship, and publication of this article.

## Funding

The authors received no financial support for the research, authorship, and publication of this article.

## Supplementary Material

ojz018_suppl_Supplementary_Appendix_A
