# Supplementary figures and images for: Five-Year Retrospective Analysis of Eurosilicone’s Silicone Gel–Filled Breast Implants
Source: Aesthet Surg J Open Forum. 2019 Jun 20;1(3):ojz018. doi: 10.1093/asjof/ojz018 (PMC7671275; doi:10.1093/asjof/ojz018)

**
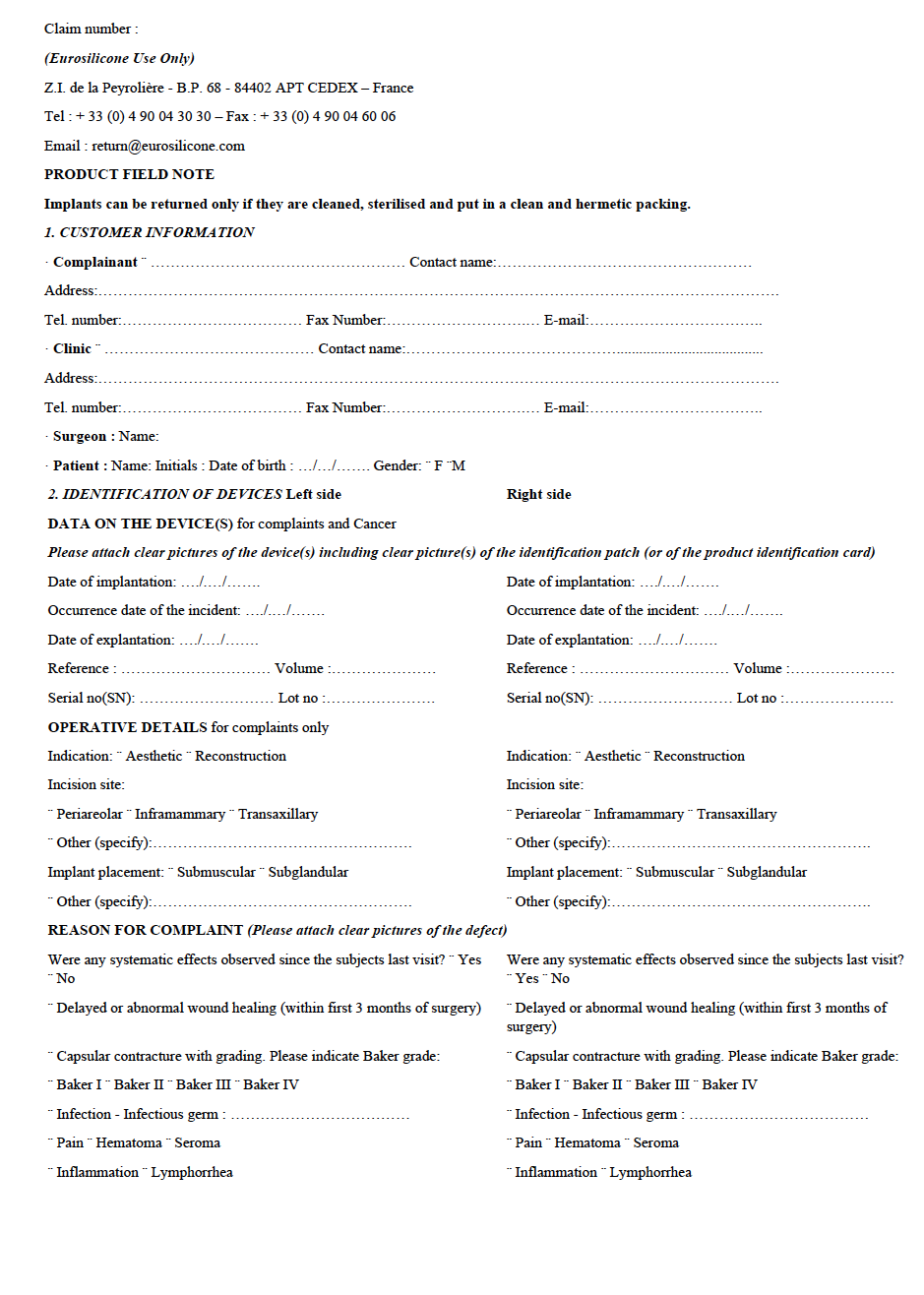
Appendix A.** Format of Complaint Form

Supplement: ojz018_suppl_Supplementary_Appendix_A [file ojz018_suppl_supplementary_appendix_a.docx]
